# The League of Mercy

**Published:** 1900-12-22

**Authors:** 


### THE LEAGUE OF MERCY.

The first annual meeting of the Presidents and Lady Presidents of the League of Mercy was held, by direction of His Royal Highness the Grand President, at St. Thomas's Hospital yesterday, in the Court Room, which was kindly lent by the Governors. There were present:—The Marquess Camden, the Earl of Dartmouth, the Earl of Clarendon, the Earl of Onslow, the Dowager Lady Lamington, the Lady Leucha Warner, the Hon. Mrs. Freeman Thomas, Isabella Lady Lennard, Lady Ellis Lady Faudel Philips, Sir Fitzroy Maclean, Bt., C.B., Lady Maclean, Sir Weetman Pearson, Bt., M.P., Mrs. Sanders, Ald. Sir William Treloar, Lady Treloar, Mr. Leigh Bennett, M.P., Mr. R. C. Antrobus, Mr. J. Allen Baker, L.C.C., Mrs. J. Allen Baker, Col. Bevington, Col. Campbell, Mr. Goddard Clarke, Miss Cons, Mrs. Seymour Corkran, Dr. E. Baxter Forman, L.C.C., Mrs. C. Tyrrell Giles, Capt. Gunton, Miss Harrington, Mr. C. T. Harris, Mrs. Harrison, Ald. John Harris, C.C., Mrs. Hodson, Col. Hughes, M.P., Mr. H. W. Keay (Mayor of Eastbourne), Mr. Thomas Lilley, Mrs. T. Buxton Morrish, Mr. Ernest Pearson, Mr. Montague Sharpe, Mrs. E. Spencer Stidolph, Mr. B. S. Straus, L.C.C., Mrs. Wightman.

There were also present:—Sir Henry Burdett, K.C.B., *Treasurer*; Lord Wolverton, Dr. W. J. Collins, Mr. J. Harrison, *Hon. Secretaries*; Col. Knollys, *Registrar*.

Letters expressing inability to attend were received from the Earl of Mansfield, Lord James of Hereford, Lord Farquhar, Lord and Lady Pirbright, Viscountess Torrington, and others.

Sir Whittaker Ellis, Bart., was the Chairman appointed by the Prince of Wales. Mr. E. A. Hambro, the Deputy-Chairman, was unavoidably absent.

Dr. W. J. Collins, one of the Hon. Secretaries, announced the pleasure of His Royal Highness that the meeting should be held and the appointment of the Chairman, Deputy-Chairman, and Hon. Secretary to the Presidents (Norman Hay Forbes, Esq., F.R.C.S.Ed.).

Sir Whittaker Ellis opened the meeting with a description of the purposes of the League. Sir Whittaker stated that although the earlier efforts of the League were contemporaneous with a period when the interests of the Empire were absorbed in the war in South Africa, it had nevertheless achieved substantial progress. Presidents had expressed some anxiety in the matter of putting forward the interests and work of the League, but he could say that the results this year augured well for the future. (Cheers.)

The first resolution was proposed by the Chairman, viz., that the following ladies and gentlemen be appointed the deputation to hand the list of subscriptions to Their Royal Highnesses the Prince and Princess of Wales:—The Grand President and Lady Grand President; H.R.H. the Duchess of Albany, the Countess of Dartmouth, the Duke of Argyll, the Marquis Camden.

The resolution was seconded by Alderman Sir William Treloar and carried unanimously.

The second resolution, that seven representative members of the Executive Council be appointed, was moved by the Marquess Camden, seconded by the Earl of Clarendon.

The following were appointed:—The Marquess Camden, the Earl of Mansfield, Lord Farquhar, Hon. Mrs. Freeman Thomas, Sir Whittaker Ellis, Bart., Mrs. Richard Lake Harrison, E. A. Hambro, Esq.

The above resolutions were carried unanimously.

Dr. Forman, L.C.C., Col. Campbell, and Mr. Straus made a few suggestions for the better organisation of the League.

Alderman John Harris, C.C., spoke of what he considered the great success in the poor district of Whitechapel, which he partly attributed to meetings he had held, one of which Dr. Collins attended, and also to the co-operation of the working men in his district.

Mr. Montague Sharpe raised the question of the participation of the local hospitals in the funds contributed to the League.

Col. Hughes, M.P., in the course of an interesting speech advocated the advisability, if possible, of some amalgamation of the various collecting funds, as he feared that the existing funds somewhat overlapped.

Sir Henry Burdett, K.C.B., Treasurer of the League, replied to the various points which had been raised by Presidents. With regard to the tickets of admission to hospitals, he pointed out that if Presidents would communicate with the hospital authorities he had no doubt that any case they might put forward would be attended to, but the letter system was practically obsolete. He hoped that hospital boards, recognising the power and influence of the Hospital Fund and the League of Mercy, would welcome representatives of these amongst them, and so establish an organic connection between the collecting agency and the institutions benefited.

Sir Henry thought that the organisation of the League might be promoted by Presidents and Lady Presidents holding social meetings or public meetings at which representatives of the Executive Council could expound the objects aimed at. He assured them that the Grand President and Lady Grand President would, as they had already done, entertain those who had worked for the League.

Sir Henry congratulated the Presidents and Lady Presidents upon the increased success of the League. Sixty-five districts had contributed an average amount of £100 apiece. The sums received were of course very much larger from some districts than others. He concluded his remarks by expressing the thanks of the Executive Council for the very kindly interest evinced by the Presidents, both those who were present and those who had written expressing their inability to attend.

It was then proposed by Mr. Antrobus, and seconded by Col. Hughes, that a sub-committee of Presidents be appointed to consider the advisability of making new regulations in accordance with the statutes.

Lord Wolverton moved a resolution expressing the thanks of the Presidents to the authorities of St. Thomas's Hospital for kindly placing the Court Room at their disposal, and a vote of thanks to Sir Whittaker Ellis for presiding at the meeting terminated the proceedings.

